# Abuse of Synthetic Cannabinoids and Cathinones in a Patient on Buprenorphine-Naloxone Treatment: A Case Report

**DOI:** 10.7759/cureus.48386

**Published:** 2023-11-06

**Authors:** Harshal Awasthi

**Affiliations:** 1 Psychiatry, Matra Chhaya Medical Center, Kanpur, IND

**Keywords:** synthetic cathinones, synthetic cannabinoids, buprenorphine treatment, toxicity, opioid agonist treatment, k2, bath salts

## Abstract

The rise of novel synthetic drugs, such as cathinones in “bath salts” and synthetic cannabinoids, poses serious health risks due to their severe side effects and unpredictable interactions with other substances, and their evasion of routine drug screenings poses additional challenges in managing opioid agonist treatments.

We present a case of an Indian male in his thirties with a history of opiate dependence who was treated with buprenorphine/naloxone. Six months into the treatment, he presented with symptoms of sedation, agitation, and paranoia. Initial toxicological screenings using enzyme-linked immunosorbent assay (ELISA) failed to detect synthetic substances, but subsequent analysis with gas chromatography-mass spectrometry (GC-MS) identified the presence of synthetic cannabinoids and cathinones. The patient admitted to using a K2 blend, unaware of its harmful constituents. This case underscores the crucial significance of meticulous monitoring in the treatment of addiction, taking into account potential interactions with synthetic substances such as K2/spice and bath salts. It highlights the necessity for individualized patient education and enhanced access to specialized toxicology testing, advocating for comprehensive strategies in addiction clinics to better identify and mitigate the risks associated with designer drugs.

## Introduction

The rise of synthetic psychoactive substances, including synthetic cannabinoids and synthetic cathinones, presents novel challenges in the realm of addiction medicine. In countries like India, where the sale of cannabis has not been legalized, these compounds, often marketed as legal alternatives to traditional illicit substances, have gained traction [1-2]. This popularity primarily stems from their ability to evade standard toxicological screening. Such capability not only fosters a misleading perception of safety but also underscores the limitations inherent in our current toxicology detection methods. The case in focus details a patient on buprenorphine/naloxone therapy who consumed a synthetic cannabinoid - 'K2' blend - which, unbeknownst to him, was adulterated with a synthetic cathinone, “bath salts.” This scenario highlights the intricacies of diagnosing such interactions, emphasizing the pressing need to enhance our detection capabilities, bolster clinical vigilance, and prioritize rigorous patient education in addiction management settings.

## Case presentation

A male patient in his thirties, with a documented history of opiate dependence, presented to our outpatient addiction clinic for a routine medication refill appointment. He was on a daily regimen of buprenorphine/naloxone (8 mg/2 mg) as part of his agonist therapy. Over the previous two months of follow-up appointments, he consistently exhibited signs of sedation. Despite these presentations, he consistently affirmed his adherence to the prescribed medication.

During one of the clinical interviews, he intermittently fell asleep, demonstrated limited engagement, and attributed this pronounced sedation to the buprenorphine/naloxone. Additionally, when awake, he appeared to be reacting to external stimuli, frequently diverting his attention to the door throughout the session. Consequently, the patient was referred to the emergency room.

In the emergency room, a thorough physical examination revealed a notable degree of conjunctival eye injection and a pulse rate of 110 beats per minute. Lungs were clear, and heart sounds were normal. Chest radiography revealed no abnormalities, and most laboratory results, including complete blood count, basic metabolic panel, liver function tests, and urinalysis, were within normal limits. His blood alcohol level was 0.0, and cultures were negative.

Further observation and testing were deemed necessary due to the unexplained paranoia and agitation. The combination of his presentation, history of opiate dependence, and observed symptoms influenced the decision to explore potential undisclosed substance abuse.

Investigations

The patient’s care in the outpatient addiction clinic continued with regular follow-up appointments. During this period, a urine toxicology screen (CEDIA [cloned enzyme donor immunoassay] and ELISA [enzyme-linked immunosorbent assay]) showed positive results for buprenorphine and negative for other illicit substances, with the patient reporting compliance with his medications.

Upon his admission to the emergency room, additional investigations were conducted, as summarized in Table 1. A chest radiography ruled out respiratory complications, showing no infiltrates. Laboratory tests, encompassing a complete blood count, basic metabolic panel, liver function tests, and urinalysis, were within normal ranges; blood and urine cultures returned negative results. A significant finding emerged from the gas chromatography-mass spectrometry testing of blood and urine samples, confirming the unexpected presence of synthetic cannabinoids and bath salts.

**Table 1:** Results of investigations CEDIA, cloned enzyme donor immunoassay; ELISA, enzyme-linked immunosorbent assay

Investigation	Findings
Urine toxicology screen (CEDIA and ELISA)	Positive for buprenorphine; negative for other substances
Chest radiography	Unremarkable
Hemoglobin	14.2 g/dL
White blood cell count	6.8 x10^3^/µL
Platelet count	225 x10^3^/µL
Sodium	140 mEq/L
Potassium	4.2 mEq/L
Glucose	95 mg/dL
Alanine aminotransferase	25 U/L
Aspartate aminotransferase	28 U/L
Bilirubin	0.8 mg/dL
Urinalysis	Clear, pH: 6.5, specific gravity: 1.020
Blood and urine cultures	Negative
Gas chromatography-mass spectrometry	Synthetic cannabinoids: 25 ng/mL; synthetic cathinones: 15 ng/mL

Outcome and follow-up

The patient was monitored for unexplained paranoia and agitation. The results of gas chromatography-mass spectrometry tests for additional agents revealed the presence of synthetic cannabinoids and bath salts. Subsequently, the patient acknowledged purchasing a K2 blend and using it regularly for the past six months. He strongly denied having used bath salts, raising the probability of the K2 blend being admixed with bath salts, which the patient himself was not aware of. He acknowledged substituting marijuana with K2, as the supplier guaranteed that it would not be detected in urine screenings. The patient’s symptoms resolved without medical intervention over the following 12 hours. In a follow-up appointment, the patient disclosed that the source of his K2 had confirmed that the admixture included bath salts with synthetic cannabinoids, intended to produce a novel high.

At the one-month follow-up, the patient reported a complete cessation of synthetic cannabinoid use. There were no observable symptoms of paranoia or agitation. He managed to return to his daily activities and work and expressed a marked improvement in his overall well-being.

During the three-month and six-month routine follow-up appointments, the patient participated in regular counseling sessions aimed at reinforcing his grasp of the dangers tied to synthetic cannabinoid use. As part of our ongoing surveillance measures, urine toxicology screens were conducted, all of which returned negative for synthetic cannabinoids. Throughout this period, the patient consistently expressed a steadfast commitment to avoiding these substances and exhibited notable progress in his recovery.

## Discussion

Bath salts (commonly sold as "Blue Silk," "Charge+," "Ivory Snow," "Ivory Wave," "Ocean Burst," "Pure Ivory," "Purple Wave," "Snow Leopard," "Stardust," "Vanilla Sky," "White Knight," and "White Lightening," "Cloud 9," "Cloud 10") [3-4] and synthetic cannabinoids (commonly sold as "Spice," "Spice Gold," "Spice Diamond," "Arctic Spice," "Silver," "Aroma," "K2," "Genie," "Scene," or "Dream") are relatively newer drugs of abuse, often collectively termed “synthetic legal intoxicating drugs” [5].

Bath salts reportedly contain cathinones, such as psychoactive chemicals. Cathinone (S)-2-amino-1-phenyl-1-propanone is a naturally occurring beta-ketone amphetamine analog found in the leaves of Catha edulis (Khat) [6]. Many bath salts contain mephedrone, mephylone, methylenedioxypyrovalerone (MDPV), or other cathinone derivatives with psychoactive properties similar to amphetamine and cocaine [7]. In particular, MDPV is a dopamine and norepinephrine reuptake inhibitor that displays potent stimulant effects [5]. Furthermore, mephedrone is suspected to act as a monoamine reuptake inhibitor, leading to the direct release of monoamines [8].

Synthetic cannabinoids contain a spectrum of synthetic chemicals that interact with cannabinoid receptors. These agents constitute four chemically distinct groups: (i) JWH compounds synthesized by John W. Huffman in the 1980s; (ii) CP compounds, a cyclohexylphenol series, developed by Pfizer in the 1970s (CP-47,497 and CP-47,497-C8); (iii) HU compounds synthesized at Hebrew University in the 1960s; and (iv) benzoylindoles, including AM-694 and RCS-4 [5].

Numerous case reports [9-18] in the existing literature underscore a varied spectrum of signs and symptoms linked to intoxication from synthetic cannabinoids and synthetic cathinones, such as bath salts, as outlined in Table 2. Given the complexity and unpredictability of these symptoms, it is imperative for clinicians to be well-acquainted with these manifestations to ensure timely and accurate identification.

**Table 2:** Reported signs and symptoms associated with the abuse of bath salts and synthetic cannabinoids

Signs and symptoms	Synthetic cathinones	Synthetic cannabinoids
Gastrointestinal	Nausea and vomiting	Nausea and vomiting
Neurological	Lightheadedness, syncope, blurred vision, myoclonus, seizures, bruxism, paresthesias	Loss of consciousness, confusion, seizures
Cardiovascular	Chest pain and palpitations, tachycardia, elevated blood pressure, diaphoresis, S-T segment changes	Tachycardia, elevated blood pressure, chest pain, cardiac ischemia
Metabolic	Hypokalemia	Hypokalemia
Autonomic	-	Fever, mydriasis
Behavioral	Severe agitation, confusion, combative behavior, paranoia, delusions and hallucinations, suicidal ideation	Agitation, anxiety, alteration of time perception, paranoia, delusions and hallucinations, dysphoria
Rare serious adverse reactions	Acute renal injury, myocardial infarction, myocarditis, methemoglobinemia, rhabdomyolysis, local tissue necrosis, disseminated intravascular coagulation, compartment syndrome, hyperthermia, multiorgan failure	-

The swift evolution of the synthetic drug market necessitates a proactive and informed response from healthcare providers, who often confront adverse reactions to poorly understood novel substances. Interdisciplinary collaboration among various medical specialists is vital for a holistic patient care approach, given the overlapping symptoms and risk of serious adverse events. It is crucial to counteract the widespread misinformation about the safety of these "legal" substances through persistent public health education and to adopt proactive strategies in clinical settings, such as judicious prescribing and comprehensive patient education, to safeguard against the dangers of synthetic drug use.

## Conclusions

The ever-changing landscape of synthetic drugs poses significant challenges for mental health professionals, as these substances often replicate the symptoms of psychiatric conditions, leading to diagnostic and treatment complexities. Healthcare providers are frequently required to address unexpected adverse effects from new synthetic substances not fully recognized in the existing medical literature. To tackle this, addiction medicine clinics must improve access to advanced drug testing tools, enabling timely and accurate identification of these drugs. This necessitates a unified approach from various specialists to ensure effective patient care in the face of overlapping symptoms and potential for severe health consequences.

Public education is vital to correct misconceptions of synthetic drugs as safe, requiring clear communication about their risks. Proactive public health messaging, alongside educational efforts, must dispel the notion propagated by vendors that these substances are legal and, by extension, safe. It is also essential to bridge the gap between the perceived legality and the actual risks these drugs pose.
